# Analysis of nutritional status and functional recovery outcomes in stroke patients under nursing care

**DOI:** 10.3389/fnut.2026.1795299

**Published:** 2026-03-30

**Authors:** Gengxiu Guo, Minxiang Wen, Zichao Xiong, Yanhua Lu

**Affiliations:** 1 Department of Neurology, Ganzhou Hospital-Nanfang Hospital, Southern Medical University, Ganzhou, Jiangxi, China; 2 Infection Management Division, Ganzhou Hospital-Nanfang Hospital, Southern Medical University, Ganzhou, Jiangxi, China; 3 Department of Rehabilitation Medicine, Ganzhou Hospital-Nanfang Hospital, Southern Medical University, Ganzhou, Jiangxi, China

**Keywords:** Barthel Index, functional independence measure, functional recovery, nursing care, nutritional status, stroke

## Abstract

**Background:**

The nutritional status is a very vital predictor of stroke patient recovery, functional independence, muscle strength, and rehabilitation outcomes. Nevertheless, little is known about the association between the state of nutrition and functional recovery during the usual nursing care in hospitalized stroke patients especially when carried out in retrospective studies.

**Objective:**

To examine the association between baseline nutritional status and functional recovery in stroke patients under hospital nursing care.

**Methodology:**

A retrospective cohort study of 500 stroke patients admitted to a tertiary teaching hospital from January 2023 to December 2025 was conducted. Nutritional status at admission was assessed using the Geriatric Nutritional Risk Index (GNRI), supported by anthropometric (BMI, mid-upper arm circumference) and functional (handgrip strength) measures. Functional recovery was evaluated via the Barthel Index (BI), Functional Independence Measure (FIM), handgrip strength, and ambulation status at admission and discharge. Multivariate linear regression adjusted for age, sex, stroke severity, comorbidities, dysphagia, and length of stay.

**Results:**

At admission, 234 patients were well-nourished, 177 at risk, and 89 malnourished. Malnourished patients were older, had higher comorbidity burdens, and lower baseline BI and FIM scores. Baseline nutritional status was significantly associated with functional improvement: well-nourished patients showed the largest gains (ΔBI 30.9 ± 9.6; ΔFIM 27.8 ± 10.1), while malnourished patients had the smallest (ΔBI 18.2 ± 8.1; ΔFIM 16.1 ± 9.0). Patients who improved their nutritional status during hospitalization achieved greater functional recovery. Multivariate regression identified serum albumin, handgrip strength, and mid-upper arm circumference as independent predictors of functional improvement.

**Conclusion:**

Baseline nutritional status strongly predicts functional recovery in hospitalized stroke patients. Routine biochemical and anthropometric measures provide practical, clinically relevant insights, emphasizing the importance of early nutritional assessment and intervention to optimize rehabilitation outcomes.

## 1 Introduction

Stroke is one of the most frequent causes of long time disability in the global community and is a significant healthcare burden because it causes functional disability and long term rehabilitation requirements. Neurological injury severity is not the only determinant of functional recovery after stroke, with modifiable systemic factors, including nutritional status, having turned out to be an important determinant of recovery patterns. The recent clinical research studies have revealed that poor nutritional condition is prevalent in stroke patients and correlates with delayed rehabilitation, dependence, and worse neurological recovery in the course of the hospitalization and post-stroke recovery. The nutritional condition can improve or deteriorate during the line of care, which emphasizes the significance of regular nutritional assessment in nursing practice, and rehabilitation centers (1).

Malnutrition is one of the most frequent effects of stroke patients due to dysphagia, neurological impairment, decreased appetite, and an accelerated metabolic rate in the post-stroke patients and prevalence of malnutrition is broadly reported in all settings (2). Recent observational studies have found that the nutritional risk factors that include GNRI, Prognostic Nutritional Index (PNI), and Controlling Nutritional status (CONT) score is highly related with poor prognosis after stroke which is manifested by lowered functional independence, low quality of neurologic recovery, and elevated mortality (3). Nutritional screening methods such as GNRI have been thus looked at as objective metrics with the capability to forecast recovery outcome and clinical complication (4).

The prevalence of malnutrition in stroke patients is quite high and in particular among older patients and those with a higher burden of comorbidity, recent cross-sectional studies have indicated that nutritional risk as measured by GNRI and other similar indices is strongly linked to poor short term recovery as measured by swallowing, mobility and daily functioning (2, 5). Research comparing the populations of rehabilitation patients suggests that nutritional impairment is associated with worse activities of daily living, slower functional improvements, which support the interplay of nutrition, physical strength, and rehabilitation efficacy in a multidimensional manner (6).

Considering the growing awareness of nutrition as a factor of post-stroke recovery, nursing personnel has a crucial role in the early detection and ongoing evaluation of nutrition. The anthropometric and biochemical measurements records offer clinical estimates of patient status during hospital stay that can be studied retrospectively as well, therefore, such records specifically allow assessing the patterns of functional recovery during normal care settings (7). Nutrition care practice studies have gone to further suggest poor outcomes to be related to malnutrition and also suggest that the practice is under-performed or under-differentiated (8).

Stroke is a major cause of long-term disability and nutritional condition is one of the most important modifiable factors in the functional recovery. Although some literature has analyzed the prevalence of malnutrition or functional outcomes individually, their combination under the framework of the organized nursing care has not been sufficiently studied. Most studies determine nutritional status just at one point, without correlating it with continued functional recovery in the course of nursing-led rehabilitation. Nurses are also a neglected part of the existing research on the nutritional status monitoring and assessment. Flaws in nutritional evaluation instruments also restrict comparability in studies. Additionally, there is limited evidence in the low and middle-income countries although the number of stroke and malnutrition is high. In clinical nutrition, it is important to distinguish between nutritional status and nutritional risk. Nutritional status refers to the patient's current measurable nutritional condition, as reflected by anthropometric, biochemical, and functional indicators. In contrast, nutritional risk represents the likelihood that a patient will experience nutrition-related adverse outcomes and is typically identified using validated screening indices such as the GNRI, CONUT, or Prognostic Nutritional Index. In the present study, nutritional risk was evaluated using GNRI as the primary standardized index, while nutritional status was described using routinely collected anthropometric, biochemical, and functional measures documented in nursing records. The objective of study is to determine the nutritional status of stroke patients undergoing the nursing care, through the use of a validated nutritional risk screening index (GNRI) together with routinely collected nutritional indicators documented in nursing assessments. It helps to determine the level of functional recovery between stroke patients based on functional independence and activities of daily living and to establish the correlation between functional recovery outcomes an4d nutritional status in stroke patients.

## 2 Methodology

### 2.1 Study design

This study was approved by the Ethics Committee of Ganzhou Hospital - Nanfang Hospital, approval number: TY-ZKY2024-006-01. The study was designed as a retrospective observational cohort study to determine the relationship between nutritional status and functional recovery outcomes in stroke patients receiving nursing care in the hospital during the hospitalization and rehabilitation. Nutritional, clinical, and functional data were taken off the medical records as part of routine nursing assessment and rehabilitation monitoring.

### 2.2 Study setting and duration

The research was conducted at the neurology and stroke rehabilitation units of a teaching hospital with a tertiary care. A review of patient records was conducted over three years from January 2023 to December 2025, involving the acute hospitalization period and the inpatient rehabilitation period. All assessments that were under analysis were incorporated in the standard nursing documentation as well as clinical care pathways.

### 2.3 Study population and sample size

500 stroke patients were analyzed. Consecutive sampling was used to select the patients who had been assessed using medical records based on the eligibility criteria. Patients whose nutritional and functional assessment was complete on both admission and discharge were only included to achieve analytical consistency.

### 2.4 Eligibility criteria

The inclusion criteria were adult patients of age 18 years and above who were diagnosed with ischemic or hemorrhagic stroke and were provided with structured nursing care and inpatient rehabilitation and those who had recorded nutritional and functional assessments. Patients were ruled out when they had incomplete records, underlying neuromuscular conditions, terminal conditions and those who were on artificial nutrition support in hospital.

### 2.5 Data collection procedure

The information was gathered using an electronic medical record and nursing chart data abstraction form. The collected demographic characteristics, type of stroke, comorbidities, nutritional indicators, and functional outcomes were used. All the data were anonymized and analyzed.

### 2.6 Nutritional status assessment

Nutritional risk at admission was primarily assessed using the Geriatric Nutritional Risk Index (GNRI), a validated and widely used nutritional risk screening tool in hospitalized and stroke populations. GNRI integrates serum albumin and weight parameters to estimate nutrition-related risk and has been shown to predict functional and clinical outcomes after stroke. Anthropometric (body mass index [BMI], mid-upper arm circumference [MUAC]) and functional (handgrip strength) parameters routinely recorded by nursing staff were used as supportive nutritional indicators to describe body composition and muscle function, but were not used independently to diagnose malnutrition. Accordingly, GNRI-defined nutritional risk groups were used for all primary comparisons of functional recovery outcomes, while individual nutritional indicators were analyzed as complementary predictors. Geriatric Nutritional Risk Index (GNRI) was calculated using the formula: (9)

GNRI = 1.489 × serum albumin (g/L) + 41.7 × (actual body weight/ideal body weight)

Patients were categorized as well-nourished (GNRI > 98), at risk of malnutrition (GNRI 92–98), or malnourished (GNRI <92). In cases where GNRI could not be calculated due to missing weight data, nutritional status was determined using hospital-specific cut-offs based on BMI, MUAC, serum albumin, and handgrip strength. Hospital-specific thresholds were defined as BMI <18.5 kg/m^2^, serum albumin <3.5 g/dl, MUAC below sex-adjusted clinical reference values, and reduced handgrip strength according to institutional nursing assessment standards. In this study, nutritional status reflects the patient's current quantified nutritional condition at admission, whereas nutritional risk represents the likelihood of future nutritional deterioration. This approach provided a comprehensive and clinically meaningful classification, capturing multiple dimensions of nutrition relevant to functional recovery during hospitalization and rehabilitation. Other indices such as CONUT or Prognostic Nutritional Index were not calculated because required variables (e.g., total cholesterol) were not consistently available in retrospective nursing records.

### 2.7 Nursing care context

Standardized nursing was given to all patients, which included attention to their dietary intake, feeding, positioning, dysphagia, and rehabilitation coordination. Routine nursing duties included nutritional observation and reassessment, and were not experimental intervention, which was in keeping with the observational quality of the study.

### 2.8 Functional recovery assessment

The evaluation of functional recovery was based on scores on admission and discharge. The Barthel Index (BI) was used to measure activities of daily living on a 0–100 scale with higher scores being more functional independence. and the Functional Independence Measure was used to measure the overall functional independence, which has motor and cognitive domains. The recovery of physical functions was also measured through changes in the handgrip strength. Other functional indicators such as the ambulation at the discharge and sit-to-stand transfer ability at the admission were obtained using the standardized nursing assessment.

### 2.9 Statistical analysis

Statistical analyses were performed using IBM SPSS Statistics for Windows, Version 26.0 (IBM Corp., Armonk, NY, USA). Continuous variables were summarized as mean ± standard deviation, while categorical variables were expressed as frequencies and percentages. Differences between nutritional status groups were assessed using one-way analysis of variance (ANOVA) for continuous variables and chi-square tests for categorical variables. Multivariate linear regression models were employed to identify independent predictors of functional recovery outcomes. They were adjusted for clinically relevant covariates, including age, sex, NIHSS at admission, comorbidities (hypertension, diabetes), dysphagia status, and length of stay. Due to the retrospective design, residual confounding may remain, and causal inferences should be made cautiously. Statistical significance was defined as a two-tailed *p*-value < 0.05.

Although the study was retrospective in design and based on available medical records, a *post hoc* power analysis was conducted to assess the adequacy of the sample size. Assuming a medium effect size for differences in functional recovery outcomes between nutritional status groups (*f* = 0.25), an alpha level of 0.05, and three comparison groups, a minimum of 252 participants was required to achieve a statistical power of 0.80, consistent with conventional statistical considerations (10). The inclusion of 500 patients in the present study therefore provided a statistical power exceeding 95%, indicating sufficient sensitivity to detect clinically meaningful differences in functional recovery outcomes between nutritional status groups.

## 3 Results

### 3.1 Study population and baseline characteristics

The retrospective review of stroke patient records was conducted on a total of 547 stroke patients. Once the ones with incomplete nutritional or functional records were filtered out, 500 records of patients were eligible and used in the final analysis. All patients included were fully assessed in terms of their nutritional status and functional outcomes at the admission and discharge through full nursing evaluation. Depending on nutritional status, the baseline demographic and clinical characteristics were significantly different. Malnourished patients were typically older and contained a higher comorbidity burden, increased hospitalization and were more severely affected by strokes at admission than their more well-nourished counterparts (Table 1).

**Table 1:** Baseline demographic and clinical characteristics (*n* = 500).

Variable	Well-nourished (*n* = 234)	At risk (*n* = 177)	Malnourished (*n* = 89)	*p*-value
Age (years)	59.8 ± 10.2	62.7 ± 10.6	66.1 ± 11.3	<0.001
Male sex, *n* (%)	140 (59.8)	101 (57.1)	52 (58.4)	0.88
BMI (kg/m^2^)	24.4 ± 3.3	22.3 ± 3.4	19.6 ± 3.1	<0.001
Ischemic stroke, *n* (%)	188 (80.3)	138 (78.0)	70 (78.7)	0.74
NIHSS at admission	8.6 ± 3.9	10.3 ± 4.2	12.8 ± 4.6	<0.001
Hypertension, *n* (%)	148 (63.2)	118 (66.7)	65 (73.0)	0.04
Diabetes mellitus, *n* (%)	91 (38.9)	82 (46.3)	45 (50.6)	0.03
Dysphagia documented, *n* (%)	46 (19.7)	54 (30.5)	41 (46.1)	<0.001
Length of stay (days)	21.4 ± 6.8	24.9 ± 7.3	28.6 ± 8.1	<0.001

Malnourished patients were found to have much higher levels of neurological impairment with more prevalence of dysphagia and also with longer hospital stays and this means they require more complex care under nursing care.

### 3.2 Nutritional status and anthropometric indicators

Nutritional indicators were shown to have a steady and gradual decrease in different nutritional categories, which supported the soundness of retrospective nursing documentation (Table 2).

**Table 2:** Comprehensive nutritional and body composition parameters at admission.

Parameter	Well-nourished	At risk	Malnourished	*p*-value
BMI (kg/m^2^)	24.4 ± 3.3	22.3 ± 3.4	19.6 ± 3.1	<0.001
Serum albumin (g/dl)	4.02 ± 0.41	3.62 ± 0.48	3.15 ± 0.46	<0.001
Hemoglobin (g/dl)	13.1 ± 1.4	12.2 ± 1.5	11.4 ± 1.6	<0.001
Total lymphocyte count (/mm^3^)	1850 ± 420	1580 ± 390	1310 ± 360	<0.001
MUAC (cm)	29.1 ± 3.2	26.7 ± 3.3	23.9 ± 3.1	<0.001
Calf circumference (cm)	34.6 ± 3.8	31.9 ± 3.7	28.8 ± 3.5	<0.001
Handgrip strength (kg)	25.1 ± 6.4	20.9 ± 5.9	17.2 ± 5.3	<0.001
GNRI score	103.6 ± 6.8	95.9 ± 7.4	87.3 ± 6.9	<0.001

**Figure 1:**
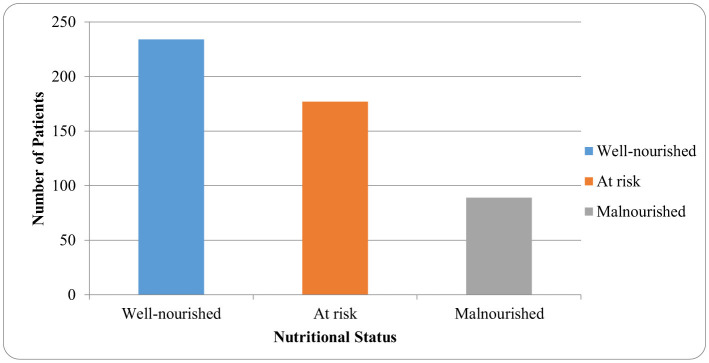
Nutritional status distribution at admission.

GNRI = 1.489 × serum albumin (g/L) + 41.7 × (actual body weight/ideal body weight). Patients were classified as well-nourished (GNRI > 98), at risk of malnutrition (GNRI 92–98), or malnourished (GNRI <92). Nutritional categories were assigned based on GNRI where available; if GNRI could not be calculated, hospital-specific cut-offs for BMI, MUAC, serum albumin, and handgrip strength were used. Reduced hemoglobin and lymphocytes in patients with malnutrition is an indication that their nutritional and immune system are impaired and this could impact on rehabilitation ability negatively (Figure 1).

### 3.3 Functional status at admission

Functional performance rates were found to be dramatically worse in malnourished patients in terms of independence and mobility in a baseline functional assessment (Table 3).

**Table 3:** Functional and physical performance status at admission.

Measure	Well-nourished	At risk	Malnourished	*p*-value
Barthel index	41.8 ± 12.7	36.2 ± 11.9	29.4 ± 10.8	<0.001
Total FIM score	63.5 ± 15.2	57.4 ± 14.6	49.6 ± 13.9	<0.001
Motor FIM	42.6 ± 10.8	37.1 ± 10.3	31.8 ± 9.6	<0.001
Cognitive FIM	20.9 ± 6.4	20.3 ± 6.1	17.8 ± 5.8	0.01
Sit-to-stand assistance required, *n* (%)	98 (41.9)	98 (55.4)	63 (70.8)	<0.001
GNRI category	Well-nourished	At risk	Malnourished	—

GNRI categories are reported to clarify how nutritional status was assigned for functional outcome comparisons. Functional outcomes are presented according to GNRI-defined nutritional categories. Nutritional impairment did not only result in a low physical independence but also the weakening of cognitive-functional level at admission.

### 3.4 Functional outcomes at discharge and magnitude of recovery

The entire groups showed an improvement by discharge but magnitude of recovery was significantly dependent on the initial nutritional status (Table 4).

**Table 4:** Functional outcomes at discharge and functional gains.

Outcome	Well-nourished	At risk	Malnourished	*p*-value
Discharge Barthel Index	72.7 ± 14.1	60.5 ± 13.6	47.6 ± 12.9	<0.001
Barthel improvement (ΔBI)	30.9 ± 9.6	24.3 ± 8.9	18.2 ± 8.1	<0.001
Discharge total FIM	91.3 ± 17.4	79.0 ± 16.2	65.7 ± 15.8	<0.001
FIM improvement (ΔFIM)	27.8 ± 10.1	21.6 ± 9.5	16.1 ± 9.0	<0.001
Independent ambulation at discharge, *n* (%)	162 (69.2)	87 (49.2)	28 (31.5)	<0.001

Patients who were well-nourished had over twice the chance of independent ambulation as the malnourished counterparts.

### 3.5 Muscle strength and physical recovery

Muscle strength recovery obtained the same trends as functional results (Table 5).

**Table 5:** Muscle strength and physical recovery indicators.

Variable	Well-nourished	At risk	Malnourished	*p*-value
Admission handgrip (kg)	25.1 ± 6.4	20.9 ± 5.9	17.2 ± 5.3	<0.001
Discharge handgrip (kg)	30.1 ± 6.8	24.6 ± 6.2	19.5 ± 5.6	<0.001
Handgrip gain (kg)	5.0 ± 2.2	3.7 ± 2.0	2.3 ± 1.7	<0.001
Pressure injury occurrence, *n* (%)	11 (4.7)	16 (9.0)	18 (20.2)	<0.001

The risk of lower muscular strength recovery and increased complication rates was found to be in the malnourished patients indicating weakness in nursing care.

### 3.6 Multivariate predictors of functional recovery

The nutritional indicators were independently related to functional recovery after the adjustment of demographic and clinical factors (Table 6).

**Table 6:** Multivariate regression analysis predicting Barthel Index improvement.

Predictor	B	95% CI	*p*-value
Serum albumin	0.33	0.26–0.41	<0.001
Baseline handgrip strength	0.27	0.20–0.35	<0.001
MUAC	0.18	0.10–0.26	0.004
GNRI score	0.16	0.08–0.24	0.01
Age	−0.15	−0.23 to −0.07	0.006
NIHSS at admission	−0.19	−0.27 to −0.11	<0.001

Recovery was predicted by both biochemical and functional nutritional markers, even though the severity of a stroke was controlled.

## 4 Discussion

The current research study investigated the relation between nutritional status and functional recovery outcome of stroke patients receiving nursing care and showed that malnutrition on admission was strongly correlated with worse neurological status, lesser functional independence, lesser muscle recovery, long hospitalization and greater complications. Notably, both biochemical and anthropometric nutritional indicators hierarchically predicted the outcomes of rehabilitation despite the correction of the clinical severity. The results and findings only strengthen the emerging evidence that the nutritional status is a key determinant of post-stroke recovery as opposed to being a secondary clinical attribute. We found that malnourished patients were much older, had worse BMI, and higher comorbidity burden, more severe strokes (higher NIHSS), more prevalent dysphagia, and were in the hospital longer. These results are consistent with the findings of Qin et al. (11), who showed that malnutrition was associated with adverse functional prognosis despite the mild stroke population, and therefore, the nutritional vulnerability enhances the neurological impairment independent of the classification of stroke severity. Physiological deterioration with age, inflammatory load, and decreased nutritional reserves are the most likely cause of poorer nutritional classes and clinical manifestations of the older population.

Malnutrition patients have a greater percentage of dysphagia which confirms the existing clinical findings. According to Wu et al. (12), the contribution of post-stroke dysphagia to hospital malnutrition was one of the highest, as it was observed that oral intake and the elevated metabolic stress caused by stroke inhibit nutritional intake and underline the importance of early nutritional monitoring by nurses. The prevalence of dysphagia in our cohort was progressively getting higher based on the type of nutritional intake that was taken, meaning that there was a bidirectional relationship between dysphagia and nutritional degradation. All the parameters of nutritional/body composition: serum albumin, hemoglobin, lymphocyte count, MUAC, calf circumference, handgrip strength, and GNRI, decreased greatly between well-nourished and malnourished groups. These results substantiate the correctness of GNRI-based categorization and are indicative of systemic nutritional deficiency of the immune, hematologic, and muscular systems.

Within the systematic review, Mancin et al. (13) highlighted the importance of utilizing multidimensional nutritional assessment with a combination of biochemical and anthropometric parameters in the acute stroke management, since the individual parameters could not adequately reflect the nutritional risk. The retrospective documentation is made to be reliable by the fact that in our study, this multidomain approach was operationalized based on nursing measures. Likewise, low albumin and hematological parameters with malnourished patients are equal to the findings by Lee et al. (14), who confirmed nutritional scoring systems in the immediate post-stroke period and showed that low nutritional indices are associated with adverse clinical recovery patterns. The gradual loss of indicators in our cohort is indicative of the fact that nutritional exhaustion of the body systems undermines the possibility of recovery. Contemporary objective phenotyping methods, such as bioelectrical impedance analysis (BIA), have been shown to independently predict functional outcomes after stroke (15). While our study relied on routinely collected anthropometric and biochemical measures documented by nursing staff, these practical assessments remain clinically informative and can complement advanced techniques in resource-limited or retrospective settings.

Patients with malnutrition had much lower Barthel Index and FIM scores at admission, which showed that they were poorer in terms of independence, mobility, and cognitive-functional performance. These findings can be compared to the results, since Di Vincenzo et al. (6) have also reported that high nutritional risk upon rehabilitation admission predicted a worse functional status and unpromising prognostic biomarkers. The nutritional deficiency is probably a cause of neuromuscular weakness, fatigue, neuroplasticity impairment so that it inhibits early rehabilitation participation. Other studies, such as Ersoy et al. (16) indicate a relationship between the level of nutritional impairment and dependency levels as it was established that there were strong correlations between nutritional scores and physical dependency among stroke patients. Our data go further to confirm this relationship by indicating that not only physical but also cognitive aspects of functional independence depends on nutritional status as indicated by lower cognitive FIM scores. Despite the fact that the functional association was witnessed in all the groups, the magnitude of recovery was much higher in well-nourished patients. Barthel Index, total FIM, and ambulation improvement were based on an evident nutritional gradient. The results in question are in line with the findings of Chen et al. (17), who found that nutritional status is a reliable predictor of stroke rehabilitation effectiveness in the context of their scoping review.

The significance of clinical importance of nutritional optimization in the early stages of patient care is demonstrated by the observed twofold higher likelihood of independent ambulation in well-nourished patients. As summarized by Kolanu et al. (18), the nutritional interventions in the hospital during rehabilitation improve the outcome of patient function by helping to support muscle protein synthesis and decreasing catabolic stress, which explains the high achievable gains in nutritionally preserved patients. Handgrip strength recovery was also associated with functional outcome, malnourished patients reported much smaller gains and pressure injuries. These outcomes are consistent with the processes of stroke-related sarcopenia that Gao and Chen (19) attributed to poor nutrition, including snowballing muscle atrophy, decreasing anabolic potential, and poor functional recovery.

The body composition has also been found to be closely linked with rehabilitation results. Irisawa and Mizushima (20) established that muscle mass and nutritional condition have a direct relationship with functional recovery in stroke rehabilitation, which aligns with our finding that less muscle reserves decrease strength gain in the face of nursing interventions and therapy. The correlation between the nutritional state and discharge independence in the study is in line with the results of Sato et al. (21), which found out that sufficient nutritional intake in the initial hospital stay is associated with the possibility of going home. The availability of energy toward intensive participation in rehabilitation is probably done by early nutritional adequacy. Also, the mechanisms of sarcopenia elaborated by Siotto et al. (22) indicate the role of insufficient nutritional intake in muscle loss and decreased recovery ability during post-stroke rehabilitation, which supports the clinical implications of anthropometric measures like calf circumference and MUAC, which we have identified.

In part, our results of less recovery in malnourished patients are consistent with those of Nozoe et al. (23), who also found that malnutrition has different effects on trunk recovery of function as compared to the strength of limbs. Although they saw selective functional effects in their study, their findings suggest general recovery impairment, which may be explained by the fact that they took into consideration several nutritional dimensions and nursing-care outcomes. In the same way, Sato et al. (24) also showed that functional recovery in the post-stroke period is inhibited by undernutrition, which confirms our finding that the predetermined nutritional deficiency limits the responsiveness to rehabilitation, even in cases of standardized clinical care.

The high complication rate of the malnourished patients especially the pressure injuries underscores the relevance of the organized nutritional nursing protocols. The results of Shabaan et al. (25) demonstrated that structured enteral feeding programs recorded a significant positive effect on the functional outcome of stroke patients, which highlights the relevance of nutritional intervention led by a nurse. Siewers et al. (26) also provide guidance reviews that further oppose the view that nutritional screening and monitoring are not to be part of regular stroke rehabilitation care pathways. In our results, we are able to confirm this recommendation by showing the measurable functional differences that are attributed to nutritional status as measured by nursing examination.

At the level of population, the evidence provided by Lu et al. (1), has indicated that nutritional status is a significant factor that does not change the outcome of stroke survivors in the long term, and our results are consistent with this finding that nutritional indicators indicate the extent of a recovery during hospitalization. Nutritional supplementation has also been found to be more effective in rehabilitation, which Liu et al. (27) synthesize interventional and provides mechanistic support to our results. The recent systematic reviews, such as that by Lang et al. (28), also support the usefulness of the oral nutritional interventions to enhance the recovery processes, implying that the functional differences in our article could be altered by means of the early intervention.

The analysis of research trends by Xie et al. (29) shows that there is growing attention to nutrition-based stroke rehabilitation across the world and it represents a paradigm shift toward multidisciplinary approaches to recovery. Our analysis is part of this developing view by showing that GNRI, albumin, MUAC, and handgrip strength have independent predictive value. The predictive power of GNRI identified in our regression analysis can be justified by Niu et al. (30), who demonstrated that the GNRI-based malnutrition risk prediction is associated with negative post-ischemic stroke complications. On the same note, the outcomes studies like Yuan et al. (31) have also identified malnutrition as a cause of high mortality in the long term, indicating that the functional deficits at an early age found in our cohort could translate into long-term mortality. Lastly, the predictive independent role of serum albumin in our multivariate analysis is consistent with that of systematic review by Wang et al. (32), who found albumin-related nutritional indices to be more powerful predictors of post-stroke cognitive and neurological outcomes.

Further clinical significance of nutritional state in stroke recovery is supported by baseline results. The lack of sex differences implies that recovery outcomes are largely associated by clinical severity and not biological sex, and the similarity in the distribution of ischemic strokes implies that it is not dependent on stroke etiology. The malnourished patients may have poorer cognitive performance due to protein-energy deficiency and poorer neuroplasticity. These findings emphasize the importance of the nutritional screening, monitoring, and personal assistance with feeding led by the nurse. The nutritional risk is also highly prevalent at admission, which also highlights the importance of nursing interventions designed to address nutrition-related issues and enhance the rehabilitation process and complications.

### 4.1 Strengths

This research has a number of strengths. First, the study involved a big sample of 500 stroke patients, hence, it had a high statistical power to indicate statistically significant relationships between the nutritional status and functional recovery outcomes. Second, the instruments that were used in the study were well-known and tested functional scales, such as the Barthel Index (BI) and Functional Independence Measure (FIM), which enabled the assessment of both physical and cognitive areas of functional functioning. Third, this method was multidimensional because of the simultaneous use of several nutritional measures, including body mass index (BMI), mid-upper arm circumference (MUAC), serum albumin and handgrip strength. Another strength that the study has is its use of routine nursing documentation as a reflection of real-life clinical practice and as offering practical information on how nutritional assessment and monitoring can potentially affect the results of rehabilitation. Lastly, analysis involved the change in the nutritional status of the patients during hospitalization, which pointed to the dynamic characteristics of nutrition and its direct effect on functional improvement.

### 4.2 Limitations

This study has several limitations. First, the retrospective design inherently restricts causal inferences, as the analysis relied on preexisting records and nursing notes, which may be variable or incomplete. Given the retrospective design and the clustering of malnutrition with older age, higher comorbidity burden, and greater stroke severity, nutritional status may serve as a marker of systemic frailty rather than an independent causal determinant of functional recovery. Reverse causation cannot be excluded, and observed associations should be interpreted as correlational rather than definitive evidence of causality. Subtle differences in documentation could have introduced bias. Second, although a composite score derived from routine nursing assessments was used, it does not replace a fully validated nutritional assessment tool, which may limit the generalizability of the findings. Third, the study was conducted in a single center, which may reduce its applicability to other healthcare settings or stroke management practices. Fourth, while multiple nutritional and functional parameters were considered, other potentially influential factors such as rehabilitation intensity, socioeconomic status, and post-discharge support were not measured and could have affected recovery outcomes. Additionally, laboratory and anthropometric data were limited to those routinely collected by nursing personnel, which may not fully capture body composition or micronutrient status. Finally, although the GNRI is a validated nutritional risk screening tool, a formal malnutrition diagnosis based on GLIM criteria could not be established due to the retrospective design and unavailability of etiologic criteria such as dietary intake or inflammation markers.

### 4.3 Future directions

To overcome these limitations, future research ought to focus on adopting prospective and multi-centered designs to prove and generalize the associations. Interventional designs may be used to test the hypothesis that nutrition-specific interventions, supported by regular nursing evaluations, may proactively improve the functional recovery of stroke patients. Also, longitudinal follow-up upon discharge should be included in order to examine the persistent effect of nutritional status on the long-term functional autonomy and quality of life. Further studies might also use more sophisticated body composition measurements, biomarkers of inflammation and measurements of rehabilitation intensity to further refine predictive models of functional recovery. Lastly, to translate these findings into a more regular clinical practice, the standardization and validation of standardized nursing protocols to assess and monitor nutrition might be necessary.

## 5 Conclusion

To sum up, this retrospective study illustrates that the nutritional status at the time of admission to the hospital is strongly linked with the functional recovery processes among stroke patients who receive nursing care. Well-nourished and improved-nutritionally-recovered patients during hospitalization recorded very high increases in the Barthel Index and Functional Independence Measure, and the enhancement of muscle strength and ambulation. The independent predictors of functional recovery were found to be serum albumin, handgrip strength, and MUAC, which increases the relevance of multidimensional nutritional assessment. These results can be used to illustrate how important regular nutritional observation and specific nursing interventions are in maximizing the recovery of stroke and give a strong case in favor of the inclusion of nutrition-oriented care into the modern stroke management plans.

## Data Availability

The raw data supporting the conclusions of this article will be made available by the authors, without undue reservation.

## References

[1] Lu HY Ho UC Kuo LT. Impact of nutritional status on outcomes of stroke survivors: a Post Hoc analysis of the NHANES. Nutrients. (2023) 15:294. doi: 10.3390/nu15020294 36678164 PMC9864300

[2] Ikenouchi H Sasaki M Johno T Yakuwa H Shiroma I Shiga T . Geriatric nutrition risk index predicts poor swallowing recovery in acute ischemic stroke. Int Med. (2025) 5708–25. doi: 10.2169/internalmedicine.5708-25 41224266 PMC13349460

[3] Tang H Gong F Guo H Dai Z Wang J Liu B . Malnutrition and risk of mortality in ischemic stroke patients treated with intravenous thrombolysis. Front Aging Neurosci. (2022) 14:834973. doi: 10.3389/fnagi.2022.834973 35264946 PMC8901046

[4] Di Vincenzo O Pagano E Cervone M Natale R Morena A Esposito A . High nutritional risk is associated with poor functional status and prognostic biomarkers in stroke patients at admission to a rehabilitation unit. Nutrients. (2023) 15:4144. doi: 10.3390/nu15194144 37836427 PMC10574786

[5] Zhu L Xia J Shao X Pu X Chen J Zhang J . The relationship between the baseline geriatric nutritional risk index (GNRI) and neurological function at the convalescence stage in patients with stroke: a cross-sectional study. BMC Geriatr. (2023) 23:173. doi: 10.1186/s12877-023-03919-w 36973674 PMC10045810

[6] Di Vincenzo O Luisi MLE Alicante P Ballarin G Biffi B Gheri CF . The assessment of the risk of malnutrition (undernutrition) in stroke patients. Nutrients. (2023) 15:683. doi: 10.3390/nu15030683 36771390 PMC9921740

[7] Hu J Liu Y Zhang Y Zhang M Zhang L. Association between nutritional status and mortality/neurological outcomes in stroke patients: a systematic review and meta-analysis. J stroke cerebrovasc dis. (2025) 34:108398. doi: 10.1016/j.jstrokecerebrovasdis.2025.108398 40675273

[8] Kim Y Lee M Mo HJ Kim C Sohn JH Yu KH . The association between malnutrition status and hemorrhagic transformation in patients with acute ischemic stroke receiving intravenous thrombolysis. BMC Neurol. (2023) 23:106. doi: 10.1186/s12883-023-03152-3 36918775 PMC10012700

[9] Bouillanne O Morineau G Dupont C Coulombel I Vincent JP Nicolis I . Geriatric nutritional risk index: a new index for evaluating at-risk elderly medical patients. Am J Clin Nutr. (2005) 82:777–83. doi: 10.1093/ajcn/82.4.777 16210706

[10] Mancin S Sguanci M Andreoli D Piredda M De Marinis MG. Nutritional assessment in acute stroke patients: a systematic review of guidelines and systematic reviews. Int J Nurs Stud. (2024) 158:104859. doi: 10.1016/j.ijnurstu.2024.104859 39043111

[11] Qin H Wang A Zuo Y Zhang Y Yang B Wei N . Malnutrition could predict 3-month functional prognosis in mild stroke patients: findings from a nationwide stroke registry. Curr Neurovasc Res. (2021) 18:489–96. doi: 10.2174/1567202619666211217130221 34923942 PMC8972270

[12] Wu Y Chen M Liu X Gao Y Li X Wang J . Prevalence and risk factors of malnutrition in elderly hospitalized patients with post-stroke dysphagia. Sci Rep. (2025) 15:39817. doi: 10.1038/s41598-025-23448-3 41233424 PMC12615810

[13] Mancin S Sguanci M Andreoli D Piredda M De Marinis MG. Nutritional assessment in acute stroke patients: a systematic review of guidelines and systematic reviews. Int J Nurs Stud. (2024) 158:104859. doi: 10.1016/j.ijnurstu.2024.104859 39043111

[14] Lee EC Jeong YG Jung JH Im Moon H. Validity of the controlling nutritional status score as a nutritional assessment tool early after stroke. Int J Rehabil Res. (2022) 45:58–64. doi: 10.1097/MRR.0000000000000503 34726196

[15] Dal Bello S Ceccarelli L Tereshko Y Gigli GL D'Anna L Valente M . Prognostic impact of malnutrition evaluated via bioelectrical impedance vector analysis (BIVA) in acute isc hemic stroke: findings from an inverse probability weighting analysis. Nutrients. (2025) 17:919. 40077787 10.3390/nu17050919 PMC11901430

[16] Ersoy S Paker N Sirin Ahisha B Kalaoglu E Kesiktaş N. The relationship between controlling nutritional status score and physical function and dependency level in stroke patients. Nutrients. (2025) 17:3734. doi: 10.3390/nu17233734 41374024 PMC12694240

[17] Chen H Fu C Fang W Wang Z Zhang D Zhang H. Influence of nutritional status on rehabilitation efficacy of patients after stroke—a scoping review. Front Neurol. (2025) 16:1502772. doi: 10.3389/fneur.2025.1502772 39944548 PMC11813746

[18] Kolanu ND Ahmed S Kerimkulova MK Stańczak M Aguirre Vera GJ Shaikh N . Influence of nutritional interventions on functional outcomes in stroke rehabilitation: a systematic review. Cureus. (2024) 16. doi: 10.7759/cureus.53711 38455777 PMC10918289

[19] Gao Z Chen H. Advances in the beneficial effects of nutrition on stroke-related Sarcopenia: a narrative review. Medicine. (2023) 102:e34048. doi: 10.1097/MD.0000000000034048 37327307 PMC10270533

[20] Irisawa H Mizushima T. Correlation of body composition and nutritional status with functional recovery in stroke rehabilitation patients. Nutrients. (2020) 12:1923. doi: 10.3390/nu12071923 32610491 PMC7400130

[21] Sato Y Yoshimura Y Abe T. Nutrition in the first week after stroke is associated with discharge to home. Nutrients. (2021) 13:943. doi: 10.3390/nu13030943 33804072 PMC8001465

[22] Siotto M Germanotta M Guerrini A Pascali S Cipollini V Cortellini L . Relationship between nutritional status, food consumption and sarcopenia in post-stroke rehabilitation: preliminary data. Nutrients. (2022) 14:4825. doi: 10.3390/nu14224825 36432512 PMC9693787

[23] Nozoe M Inoue T Ishida M Yamamoto K Kanai M. Malnutrition on admission is associated with trunk function recovery but not with lower limb muscle strength recovery in patients with acute stroke: an observational cohort study. Nutrition. (2023) 109:111971. doi: 10.1016/j.nut.2023.111971 36745968

[24] Sato K Inoue T Maeda K Shimizu A Ueshima J Ishida Y . Undernutrition at admission suppresses post-stroke recovery of trunk function. J Stroke Cerebrovasc Dis. (2022) 31:106354. doi: 10.1016/j.jstrokecerebrovasdis.2022.106354 35176691

[25] Shabaan G Abdelwarith M Diab M. Enteral feeding protocol to improvement functional outcome in stroke patient. Egypt j health care. (2021) 12:735–45. doi: 10.21608/ejhc.2021.149446

[26] Siewers K Svaerke K Rosenørn AE Christensen H. Nutritional care in rehabilitation and acute care of stroke patients: a systematic review of clinical practice guidelines. Front Stroke. (2025) 4:1558019. doi: 10.3389/fstro.2025.1558019 41541884 PMC12802607

[27] Liu J Dong J Guo J. The effects of nutrition supplement on rehabilitation for patients with troke: analysis based on 16 randomized controlled trials. Medicine. (2022) 101:e29651. 36123946 10.1097/MD.0000000000029651 PMC9478301

[28] Lang A Álvarez-Martínez M Zariwala MG Ganesh A Geva S Ray S . Oral nutritional interventions for stroke recovery: a systematic review. Nutr Rev. (2025) 179. doi: 10.1093/nutrit/nuaf179 41066148

[29] Xie Y Xiong Y Sun M Zhao Y Wu M. Research trends in nutritional interventions for stroke: a bibliometric analysis and literature review. Front Nutr. (2024) 11:1489222. doi: 10.3389/fnut.2024.1489222 39483787 PMC11526124

[30] Niu M Zhang F Wang L Yang H Zhu L Song S. Association of malnutrition risk evaluated by the geriatric nutritional risk index with post-stroke myocardial injury among older patients with first-ever ischemic stroke. BMC Geriatr. (2025) 25:140. doi: 10.1186/s12877-025-05796-x 40025439 PMC11872321

[31] Yuan K Zhu S Wang H Chen J Zhang X Xu P . Association between malnutrition and long-term mortality in older adults with ischemic stroke. Clin Nutr. (2021) 40:2535–42. doi: 10.1016/j.clnu.2021.04.018 33932800

[32] Wang YQ He X Huang XL Qin FL Mao FL Cheng YM . The role of serum albumin and albumin-related nutritional indices in predicting post-stroke cognitive impairment: a systematic review and meta-analysis. Front Neurol. (2025) 16:1641711. doi: 10.3389/fneur.2025.1641711 40881792 PMC12382350

